# Autosomal recessive non‐syndromic hearing loss genes in Pakistan during the previous three decades

**DOI:** 10.1111/jcmm.18119

**Published:** 2024-03-27

**Authors:** Madiha Shadab, Ansar Ahmed Abbasi, Ahsan Ejaz, Afif Ben‐Mahmoud, Vijay Gupta, Hyung‐Goo Kim, Barbara Vona

**Affiliations:** ^1^ Department of Zoology Mirpur University of Science and Technology Mirpur Pakistan; ^2^ Department of Physics University of Kotli Azad Jammu and Kashmir Kotli Pakistan; ^3^ School of Nuclear Science and Technology Lanzhou University Lanzhou China; ^4^ Neurological Disorders Research Center, Qatar Biomedical Research Institute, Hamad Bin Khalifa University Doha Qatar; ^5^ College of Health & Life Sciences Hamad Bin Khalifa University (HBKU) Doha Qatar; ^6^ Institute of Human Genetics University Medical Center Göttingen Göttingen Germany; ^7^ Institute for Auditory Neuroscience and Inner Ear Lab University Medical Center Göttingen Göttingen Germany

**Keywords:** genetic counselling, genetic epidemiology, genotype, non‐syndromic hearing loss, Pakistani population, phenotype

## Abstract

Hearing loss is a clinically and genetically heterogeneous disorder, with over 148 genes and 170 loci associated with its pathogenesis. The spectrum and frequency of causal variants vary across different genetic ancestries and are more prevalent in populations that practice consanguineous marriages. Pakistan has a rich history of autosomal recessive gene discovery related to non‐syndromic hearing loss. Since the first linkage analysis with a Pakistani family that led to the mapping of the DFNB1 locus on chromosome 13, 51 genes associated with this disorder have been identified in this population. Among these, 13 of the most prevalent genes, namely *CDH23*, *CIB2*, *CLDN14*, *GJB2*, *HGF*, *MARVELD2*, *MYO7A*, *MYO15A*, *MSRB3*, *OTOF*, *SLC26A4*, *TMC1* and *TMPRSS3*, account for more than half of all cases of profound hearing loss, while the prevalence of other genes is less than 2% individually. In this review, we discuss the most common autosomal recessive non‐syndromic hearing loss genes in Pakistani individuals as well as the genetic mapping and sequencing approaches used to discover them. Furthermore, we identified enriched gene ontology terms and common pathways involved in these 51 autosomal recessive non‐syndromic hearing loss genes to gain a better understanding of the underlying mechanisms. Establishing a molecular understanding of the disorder may aid in reducing its future prevalence by enabling timely diagnostics and genetic counselling, leading to more effective clinical management and treatments of hearing loss.

## 1 INTRODUCTION

Pakistan, home to a population of 232 million according to the February 2023 United Nations census, ranks as the fifth most populous country in the world. The country's population is dispersed across multiple provinces, each with unique ethnic and linguistic diversity backgrounds. Consanguineous marriage is prevalent in most communities, making Pakistan one of the countries with the highest consanguinity rates globally. Consequently, homozygous pathogenic alleles steadily accumulate, leading to an ultimate increase in the prevalence of autosomal recessive disorders such as congenital hearing loss (HL). Out of the estimated 14.5 million Pakistanis living with HL, about half have a genetic aetiology.^1^ According to the World Health Organization, more than 1.5 billion people around the world are impacted by HL, and roughly 28% of them, or 430 million individuals, have a disabling level of HL. This is concerning because the number of people with disabling HL is projected to exceed 700 million by 2050 (https://www.who.int/health‐topics/hearing‐loss).

Although individuals from the Pakistani community represent a minority of patients tested within the Asian population,^2^ their contributions have yielded valuable insights into the molecular genetics and biology of hearing and deafness that hold relevance across diverse global genetic backgrounds. While this review does not aim to provide an exhaustive catalogue of all the variants identified in the Pakistani population, its goal is to highlight the exciting discoveries that have enriched our current understanding of the genetic epidemiology of HL. Although many genes have been implicated in HL (with many more yet to be discovered) in the Pakistani population, a dozen or so genes appear to play a predominant role in its genetic makeup. In this review, we provide a brief overview of the clinical heterogeneity and history of HL gene discovery, analyse gene ontology (GO) terms and pathways associated with HL and expand upon the key genes that explain a significant proportion of HL in the Pakistani population.

## 2 CHARACTERISTICS AND CLINICAL CLASSIFICATIONS FOR HEARING LOSS

HL, the permanent loss of hearing ability, encompasses several key characteristics.^3^ Firstly, the anatomical site of the cochlear defect is used to distinguish between conductive or sensorineural HL (SNHL), with the latter being most prevalent.^4^ Conductive HL occurs due to abnormalities in the outer or middle ear that hinder sound conduction, while SNHL results from damage to the inner ear or auditory nerve. When both conductive and sensorineural components are present, mixed HL ensues. Additionally, abnormalities or malfunctions beyond the cochlea, such as in the eighth cranial nerve, auditory brainstem, or cortex, lead to central auditory impairment.^5^ The second characteristic pertains to the age of onset relative to speech development.^3^ Pre‐lingual HL manifests before the critical period for speech development, including congenital onset or HL already present at birth. Post‐lingual HL, on the other hand, includes individuals with onset following the critical period for speech acquisition, spanning from childhood to advanced age and includes age‐related HL.^3^ The third feature relates to the degree of HL and the associated hearing thresholds used to define severity levels, which include mild (hearing threshold 20–40 dB), moderate (41–55 dB), moderately severe (56–70 dB), severe (71–90 dB) and profound (90 dB).^3^ HL can affect low (<1000 Hz), mid (1000–2000 Hz) or high (>2000 Hz) frequencies. Classifying HL based on these factors is crucial for determining the most appropriate treatment and management strategies for each individual. The fourth attribute of HL concerns the presence or absence of progression, which is determined by serial audiometry. Although most late‐onset HL typically follows a progressive course, the majority of individuals with congenital HL have a non‐progressive form. HL can affect one or both ears, referred to as unilateral or bilateral, respectively. Asymmetric HL is another possibility where both ears are affected to differing degrees.^6^ The sixth feature is whether HL is part of a syndrome that involves concomitant abnormalities in additional organ systems or is the sole presenting feature, as seen with non‐syndromic HL (NSHL), which is observed in the vast majority of congenital cases.^3^

Inheritance patterns are another defining characteristic of HL. Most hereditary HL exhibits an autosomal recessive inheritance, accounting for around 75% of congenital cases,^7^ while roughly 18%, 1%–3% and 1% of NSHL are caused by autosomal dominant, X‐linked and mitochondrial forms, respectively.^8^ Those with autosomal recessive non‐syndromic hearing loss (ARNSHL) inherit either a single heterozygous variant from each asymptomatic parent, as observed for homozygous variants, or receive two distinct variants in the same gene, one from each parent, known as compound heterozygous variants. If both parents carry a single disease allele on the same gene, their offspring have a 25% chance of inheriting two disease alleles to develop HL, a 50% chance of inheriting one disease allele to be an asymptomatic carrier and a 25% chance of inheriting no disease alleles and being unaffected.

## 3 METHODS USED FOR IDENTIFICATION OF ARNSHL GENES

**FIGURE 1:**
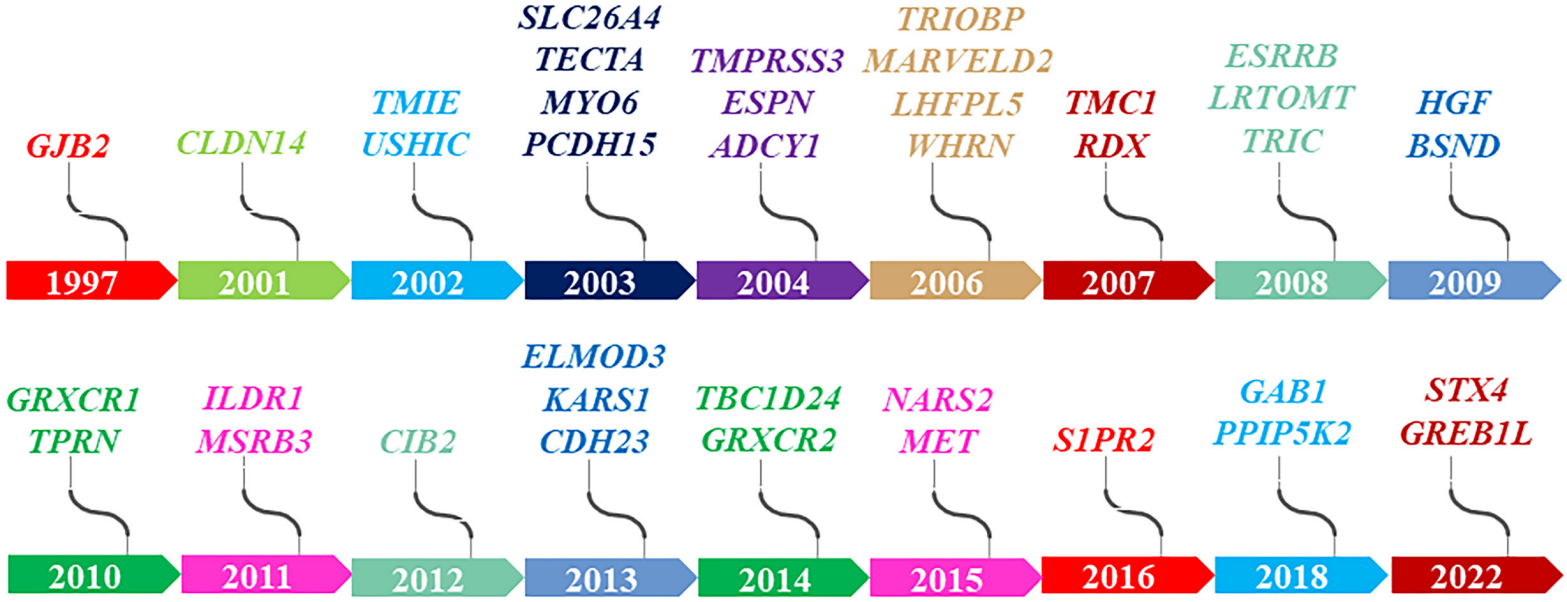
Timeline of 39 ARNSHL genes identified by collaborating with the Pakistani population over the past 25 years.

Over the past three decades, there has been significant progress in identifying genes and loci associated with HL in Pakistani families (see Table 1 and Figure 1), using a range of evolving technologies. One method used to locate Mendelian disease loci involves classic parametric linkage analysis, which utilizes genetic STRP markers or SNP maps along with information about mode of inheritance, penetrance and allele frequencies.^9^ This approach is based on the principle that chromosomal regions near genetic variants segregate together during meiosis. By analysing nearby markers, a genomic locus carrying a causative mutation may be statistically linked to the disease. The strength of the linkage evidence (or lack thereof) is assessed using the estimation of a logarithm of the odds (LOD) score. A LOD score greater than 3.0 is considered significant for linkage.^10^ Once one or more potentially significant linked intervals have been identified through linkage analysis, the next step involves candidate gene sequencing of all genes in the region. Thirty‐nine ARNSHL genes have been identified in the Pakistani population with the support of linkage analysis. Homozygosity mapping is considered the best method for uncovering chromosomal regions that may harbour a causal variant in consanguineous families.^11^ This method involves examining genotypes from multiple affected and unaffected family members to identify the number and size of runs of homozygosity. This information can be used to narrow down the genomic regions that are likely to contain the disease‐causing variant. In genetically heterogeneous disorders, homozygosity mapping can reduce the need to sequence various candidate genes and instead focus on specific genomic regions.^12^ Twelve ARNSHL genes have been identified by using homozygosity mapping.

**TABLE 1:** Fifty‐one ARNSHL genes were found mutated in the Pakistani population in the last 25 years listed in chronological order.

Gene symbol	DFN locus	Chr position	Gene name	MIM	Discovery method	Onset and severity	Reference
*GJB2*	DFNB1	13q12.11	Gap junction protein beta 2	220290	LA	Prelingual, severe to profound	[13]
*MYO15A*	DFNB3	17q11.2	Myosin XVA	600316	NGS	Congenital, severe to profound	[14]
*CLDN14*	DFNB29	21q22.1	Claudin 14	614035	LA	Congenital, profound	[15]
*TMIE*	DFNB6	3p21.31	Transmembrane inner ear	600971	LA	Prelingual, severe to profound	[16]
*TMC1*	DFNB7/11	9q21.13	Transmembrane channel like 1	600974	LA	Prelingual, severe to profound, and vestibular dysfunction	[17]
*USH1C*	DFNB18	11p15.1	Greb1‐like retinoic acid receptor coactivator	602092	LA	Prelingual, moderate to severe	[18]
*PCDH15*	DFNB23	10q21.1	Protocadherin‐related 15	609533	LA	Prelingual, profound	[19]
*TECTA*	DFNB21	11q23.3	Tectorin alpha	603629	LA	Prelingual, moderate to severe	[20]
*SLC26A4*	DFNB4	7q22.3	Solute carrier family 26, member 4	600791	HM, LA	Prelingual, severe to profound	[21]
*MYO6*	DFNB37	6q14.1	Myosin VI	607821	LA	Congenital, profound	[22]
*TMPRSS3*	DFNB8/10	21q22.3	Transmembrane serine protease 3	614861	LA	Congenital, profound	[23]
*ESPN*	DFNB36	1p36.3	Espin	609006	HM, LA	Congenital, profound, with or without vestibular dysfunction	[24]
*WHRN*	DFNB31	9q32	Cask‐interacting protein	607084	LA	Congenital, profound	[25]
*TRIOBP*	DFNB28	22q13.1	TRIO and F‐actin‐binding protein	609823	LA	Congenital, profound	[26]
*MARVELD2*	DFNB49	5q13.2	MARVEL domain containing 2	610153	LA	Prelingual, Moderate to severe	[27]
*LHFPL5*	DFNB67	6p21.31	LHFPL tetraspan subfamily member 5	610265	LA, HM	Prelingual, severe to profound	[28]
*RDX*	DFNB24	11q22.3	Radixin	611022	LA	Pre‐lingual, profound	[29]
*ESRRB*	DFNB35	14q24.3	Oestrogen‐related receptor beta	608565	LA	Prelingual, severe to profound	[30]
*LRTOMT*	DFNB63	11q13.4	Leucine‐rich transmembrane and O‐methyltransferase domain containing	611451	LA	Congenital, profound	[31]
*OTOF*	DFNB9	2p23.3	Otoferlin	601071	LA	Prelingual, severe‐to‐profound	[32]
*HGF*	DFNB39	7q21.11	Hepatocyte growth factor	608265	LA	Prelingual, severe to profound	[33]
*BSND*	DFNB73	1q32.3	Barttin CLCNK type accessory subunit beta	602522	LA	Prelingual, profound	[34]
*TPRN*	DFNB79	9q34.3	Taperin	613307	NGS / LA	Prelingual, severe to profound	[35]
*GRXCR1*	DFNB25	4p13	Glutaredoxin and cysteine‐rich domain containing 1	615837	HM, LA	Congenital, moderate to profound	[36]
*MSRB3*	DFNB74	12q14.3	Methionine sulfoxide reductase B3	613718	LA	Prelingual, severe to profound	[37]
*GIPC3*	DFNB15/72/95	19p13.3	GIPC PDZ domain containing family member 3	601869	LA	Prelingual, mild to profound	[38]
*ILDR1*	DFNB42	3q13.33	Immunoglobulin‐like domain containing receptor 1	609646	LA	Prelingual, profound	[39]
*PJVK*	DFNB59	2q31.2	Pejvakin	610220	LA, HM	Prelingual, profound	[40]
*CIB2*	DFNB48	15q25.1	Calcium‐ and integrin‐binding family member 2	609439	LA		[41]
*CDH23*	DFNB12	17q12	Cadherin 23	601386	ES	Congenital, Profound	[42]
*KARS1*	DFNB89	16q23.1	Lysyl‐tRNA synthetase 1	613916	LA, ES	Prelingual, profound	[43]
*OTOA*	DFNB22	16p13.1‐q11.2	Otoancorin	607039	HM	Congenital, moderate to severe	[44]
*ELMOD3*	DFNB88	2p11.2	ELMO domain containing 3	615429	ES, HM	Prelingual, severe to profound	[45]
*GRXCR2*	DFNB101	5q32	Glutaredoxin and cysteine‐rich domain containing 2	615837	LA, HM, ES	Congenital, severe to profound	[46]
*TBC1D24*	DFNB86	16p13.3	TBC1 domain family member 24	614617	ES, LA	Prelingual, profound	[47]
*ADCY1*	DFNB44	7p12.3	Adenylate cyclase 1	610154	LA	Congenital, Severe to profound	[48]
*NARS2*	DFNB94	11q14.1	Asparaginyl‐tRNA synthetase 2, mitochondrial	618434	ES, LA	Prelingual severe to profound	[49]
*MET*	DFNB97	7q31.2	MET proto‐oncogene, receptor tyrosine kinase	164860	LA, HM, ES	Congenital, severe	[50]
*S1PR2*	DFNB68	19p13.2	Sphingosine‐1‐phosphate receptor 2	608565	ES, LA, HM	Congenital, profound	[51]
*EPS8L2*	DFNB102	11p15.5	EPS8 like 2	617637	ES, GS	Post lingual, severe	[52]
*PDZD7*	DFNB57	10q24.31	PDZ domain containing 7	618003	ES	Congenital, severe to profound	[53]
*PPIP5K2*	DFNB100	5q21.1	Diphosphoinositol pentakisphosphate kinase 2	618422	ES, HM, LA	Prelingual, severe to profound	[54]
*GAB1*	DFNB26	4q31.21	GRB2‐associated binding protein 1	605429	LA, ES	Congenital, severe to profound	[55]
*TMEM132E*	DFNB99	17q12	Transmembrane protein 132E	618481	ES	Prelingual, profound	[56]
*LOXHD1*	DFNB77	18q21.1	Lipoxygenase homology PLAT domains 1	613079	ES	Congenital, profound	[57]
*CABP2*	DFNB93	11q13.2	Calcium‐binding protein 2	614899	ES	Prelingual, moderate to severe	[58]
*SERPINB6*	DFNB91	6p25.2	Serpin family B member 6	613453	ES	Congenital, profound	[59]
*PTPRQ*	DFNB84	12q21.31	Protein tyrosine phosphatase receptor type Q	613391	ES, LA	Prelingual, severe to profound	[59]
*SIX5*	ND	19q13.32	SIX homeobox 5	610896	ES	Congenital and post‐lingual, severe to profound	[60]
*STX4*	ND	16p11.2	Syntaxin 4	186591	ES, HM, LA	Congenital, profound	[61]
*GREB1L*	ND	18q11.1‐q11.2	GREB1 Like Retinoic Acid Receptor Coactivator	619274	ES	Congenital, profound	[62]

Abbreviations: ES, exome sequencing; GS, genome sequencing; HM, homozygosity mapping; LA, linkage analysis; ND, not defined; NGS, next‐generation sequencing.

Gene discovery has been further revolutionized by advances in exome and genome sequencing. Exome sequencing targets the protein‐coding portion of genes, representing only about 1% (30 Mb) of the genome but capturing 85% of the disease‐causing mutations in Mendelian disorders.^63^ Consequently, sequencing the entire exome has become a powerful tool for identifying disease‐associated variants, including monogenic disorders like HL as well as predisposing variants in common diseases. This has led to faster identification of such variants.^64^ Genome sequencing provides a comprehensive view of an individual's entire genome, including both coding and non‐coding regions, allowing for the identification of both rare and common variants associated with HL. In recent years, genome sequencing has enabled the discovery of new HL genes, particularly when variants reside in non‐coding regions that were previously overlooked.^65^ Using exome and genome sequencing, 22 gene variants in novel NSHL‐associated genes in the Pakistani population have been identified. Some of these 22 genes (12 genes) were also identified using other techniques described above such as linkage analysis and homozygosity mapping (as mentioned in Table 1). Moreover, it has facilitated the identification of pathogenic variants in known genes and enables the assessment of structural variations, such as copy number variations and structural rearrangements that can contribute to HL. As sequencing technologies continue to advance and become more cost‐effective, genome sequencing is likely to become an increasingly valuable tool for HL gene discovery and expedite the development of effective therapies.

In the Pakistani population, variants in a total of 51 genes have been implicated in ARNSHL through the approaches described above (Table 1). Owing to the unique genetic background and high consanguinity rate of the Pakistani population, 39 of these 51 genes have been discovered between 1997 and 2022 (Figure 1), underscoring the significant contribution of the Pakistani population to unravelling the genetics of HL.

Although next‐generation sequencing‐based molecular tests are still in their infancy, they have shown clinical value for single‐gene diseases. The full potential of exome and genome sequencing will enrich genomic medicine beyond rare single‐gene disorders. Further developments in next‐generation sequencing technologies and bioinformatics tools will enhance data analysis and clinical extraction. Recent studies justify the effort and expense of incorporating these innovative ideas into molecular diagnostics practice. While advances in next‐generation sequencing have sped up the detection of disease‐causing variants, they cannot overcome universal limitations faced in Mendelian disorders. Exploration of the genetics of ARNSHL is optimal with large pedigrees that ideally support gene mapping approaches and next‐generation sequencing. Furthermore, identification of variants in compound heterozygosity is critical for validating genetic findings. Variants with limited evidence to conclusively assign likely pathogenic or likely benign classifications remain a great challenge in genomic medicine in general. Variable expressivity can affect interpretation as well as outcomes, as not everyone who harbours particular variants will develop the disease due to possible incomplete penetrance. It is critical to use these technologies in collaboration with various patient populations, genetic counsellors, and medical geneticists. This collaborative endeavour will be crucial for increasing our understanding of the genetic basis of HL and discovering novel therapies to treat it.

## 4 GENE ONTOLOGIES AND PATHWAYS OF RECURRENT ARNSHL GENES IN PAKISTAN

By conducting molecular genetic studies on consanguineous pedigrees, researchers have been able to identify deleterious alleles in 51 out of the 68 genes linked to ARNSHL, thanks in part to participation of Pakistani families with HL.^66^ Notably, among the 51 autosomal recessive genes we describe, nine genes (*GJB2*, *TECTA*, *MYO6*, *ESPN*, *TMC1*, *TPRN*, *ELMOD3*, *TBC1D24* and *PTPRQ*) have been associated with an autosomal‐dominant inheritance pattern as well. While the identification of 51 genes associated with HL may seem daunting, researchers can simplify the complexity by examining the established metabolic and cell signalling pathways through which these genes interact in various organs or by identifying enriched ontology clusters. One of the fundamental presumptions of network analysis and ontology clustering is that there are functional connections between genes whose malfunction leads to disease manifestation. Understanding biological systems, disease states and the mechanisms by which drugs affect them relies on identifying these common pathways and ontology terms. Therefore, by recognizing the shared biological pathways involved in HL and other disorders, researchers can gain insights into the underlying causes of these diseases and develop effective therapeutic interventions.

**FIGURE 2:**
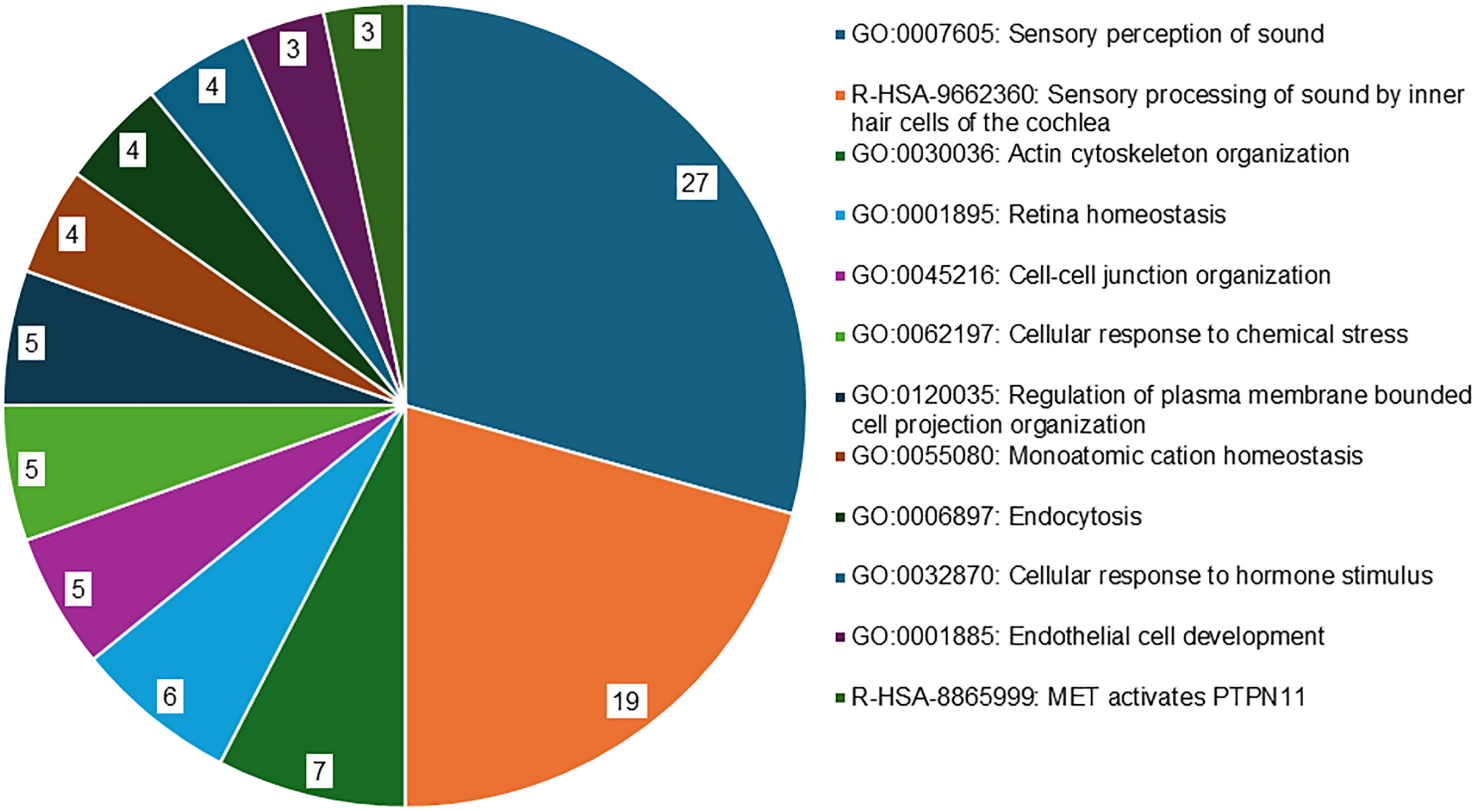
Pie chart showing the results of Metascape analysis of enriched gene ontology (GO) clusters. The chart displays the top 12 clusters and their representative enriched terms (one per cluster). The ‘Count’ refers to the number of genes in our list of 51 genes associated with ARNSHL that are included in the given ontology term. To classify the genes associated with ARNSHL according to their function, we performed pathway and process enrichment and network analysis using Metascape (http://metascape.org). Using this approach, we identified the enrichment of GO terms and genes related to various biological pathways.

To gain further insights into the functional roles of the 51 ARNSHL‐associated genes, gene enrichment analysis and MCODE algorithm were employed using Metascape.^67^ The annotation, enrichment and genes used to perform the Metascape analysis are shown in Table S1. Enrichment analysis using Metascape identified four areas of enriched protein clusters where proteins are closely related based on similar pathways involved. The first cluster of genes was involved in sensory perception of sound (27 genes, GO:0007605), the second cluster in sensory processing of sound by cochlear inner hair cells (IHCs) (19 genes, R‐HSA‐9662360), the third cluster in actin cytoskeleton organization (7 genes, GO:0030036), and the fourth cluster in retina homeostasis (6 genes, GO:0001895) (Figure 2). To facilitate the visualization of the results, we created a pie chart using Microsoft Excel software to illustrate the count of genes belonging to each category of enriched GO terms.

Protein clustering also revealed that the 51 genes associated with ARNSHL regulate several common biological pathways. These pathways include cell–cell junction organization (5 genes, GO:0045216), cellular response to chemical stress (5 genes, GO:0062197) and regulation of plasma membrane‐bounded cell projection organization (5 genes, GO:0120035). Other biological processes involved were monoatomic cation homeostasis (4 genes, GO:0055080), endocytosis (4 genes, GO:0006897), cellular response to hormone stimulus (4 genes, GO:0032870), endothelial cell development (3 genes, GO:0001885) and MET activates PTPN11 (3 genes, R‐HSA‐8865999) (Figure 2). ‘MET activates PTPN11’ refers to a signalling pathway that involves the interaction of two proteins of MET and PTPN11. This pathway has been implicated in a variety of cellular processes and diseases, including cancer and developmental disorders.

Overall, our bioinformatics analysis highlights the intricate role of ARNSHL genes in various cellular signalling pathways and biological processes. These findings have important implications for understanding the underlying mechanisms of ARNSHL and for the development of targeted therapies aimed at restoring normal function to disrupted pathways.

## 5 RECURRENT GENES AND VARIANTS ASSOCIATED WITH HEARING LOSS IN THE PAKISTANI POPULATION

Despite the extensive genetic heterogeneity in ARNSHL, a few genes account for over half of the cases. One large cohort study of Pakistani patients identified variants in six genes (*GJB2*, *HGF*, *MYO7A*, *SLC26A4*, *CIB2* and *TMC1*) that collectively explained up to 57% of recessively inherited severe to profound HL.^66^ Another cohort study found pathogenic or possibly deleterious mutations in *GJB2*, *MYO7A*, *CDH23* and *MYO15A* in 13 out of 21 (61.9%) consanguineous Pakistani families with HL.^68^ This part of the review aims to shed light on the most prevalent genes and variants in the Pakistani population. By providing valuable insights into the molecular genetics and epidemiology of ARNSHL, this information can be utilized to develop effective genetic screening and counselling programs for individuals and families affected by this condition and also develop personalized treatment strategies for the affected individuals.

### 5.1 CDH23

*CDH23* (DFNB12, USH1D) encodes cadherin 23, a crucial component of the extracellular filaments responsible for formation and function of the mechanosensory hair bundles of the inner ear.^69^ The hair bundle consists of stereocilia, hair‐like projections that respond to vibration of the basilar membrane and stimulate hair cells by depolarization.^70^ *CDH23* is expressed not only in the cochlear hair cells but also in the vestibular hair cells and the photoreceptor layer of the retina.^71^ In fact, *CDH23* is one of the most highly expressed genes in the vestibular hair cells, where it plays a crucial role in maintaining their mechanical and electrophysiological properties. Different isoforms of *CDH23* have tissue‐specific functions. Biallelic variants disrupting *CDH23* cause ARNSHL (DFNB12) and Usher syndrome type I (USH1D), characterized by congenital SNHL, vestibular dysfunction and early onset retinitis pigmentosa.^72^ Although *CDH23*‐associated NSHL has traditionally been associated with congenital onset and profound severity, recent studies have revealed a broad phenotypic spectrum that includes individuals with biallelic variants presenting HL in the second to seventh decade of life, expanding the understanding of DFNB12.^73^ As one of the most common genetic causes of HL, many *CDH23* pathogenic alleles have been identified in affected individuals of Pakistani ancestry, contributing to up to 5% of NSHL and USH1D^1^ due to numerous pathogenic alleles of *CDH23.* ^66^ The c.2968G>A, p.(Asp990Asn) and c.6133G>A, p.(Asp2045Asn) alleles are major causes of *CDH23*‐associated deafness in Pakistan but the most commonly occurring variant of these genes is c.6050‐9G>A (splicing).^66^, ^74^ At least 28 additional *CDH23* variants have been identified in different ethnic groups in Pakistan. However, so far no experimental evidence shows an impact on protein function.^66^, ^74^, ^75^ A comprehensive understanding of disease mechanisms in *CDH23*‐associated disorders may significantly impact the diagnosis and management of HL and deaf‐blindness worldwide.

### 5.2 CIB2

Calcium‐ and integrin‐binding protein 2 (CIB2) is a crucial component of the mechano‐transduction (MET) process in cochlear hair cells, demonstrated by the complete abolishment of MET currents upon genetic disruption.^76^, ^77^ MET is the process by which sound vibrations are converted into electrical signals that the brain can interpret as sound. CIB2 is an elongation factor‐hand domain‐containing protein that binds Ca^2+^ ions and has been associated with various functions, including integrin signalling in platelets and skeletal muscle and autophagy, indicating its considerable functional flexibility.^78^ Although its clinical presentation seems to be limited to hearing deficits, typically manifesting as bilateral, profound, pre‐lingual HL, *CIB2* has been linked to a wide range of functions beyond hearing physiology. A brief association with Usher syndrome was eventually refuted.^41^, ^79^ A large Pakistani cohort study has estimated the prevalence of *CIB2*‐associated HL as 8.6%.^66^ The homozygous recurrent c.272T>C, p.Phe91Ser (NM_006383.3) variant is the major cause of NSHL in the Pakistani community and has been identified in 81 families.^41^ Two additional rare variants have been identified in affected Pakistani individuals: c.192G>C, p.Glu64Asp and c.297C>G, p.Cys99Trp.^41^

### 5.3 CLDN14

*CLDN14*, also known as DFNB29, encodes the claudin‐14 protein, an essential membrane protein forming tight junctions in the inner ear.^15^ These tight junctions are crucial for compartmentalizing the endolymphatic and perilymphatic fluid compartments, maintaining cell polarity, and regulating intercellular permeability to solutes, ions, and water.^80^ *CLDN14* is expressed in the inner and outer hair cells, supporting cells, and Reissner's membrane.^81^ It plays a crucial role in maintaining ion homeostasis and calcium levels in the endolymph and perilymph fluids, which is essential for the MET process of cochlear hair cells.^82^ MET is initiated by the opening of cation channels near the tip of the stereocilia and requires ion homeostasis to maintain ion gradients for preservation of the endocochlear potential.^80^ While the endocochlear potential in *Cldn14‐*null mice has been determined as normal, rapid degeneration of outer hair cells and progressive slower degeneration of IHCs is thought to be due to the compromised tight junction barrier.^83^ In Pakistani families, seven different variants of *CLDN14* are responsible for 2%–3.3% of cases of profound deafness or moderate to severe HL^66^, ^84^ with the most prevalent variant being c.254T>A, p.Val85Asp (NM_001146079.2) identified in 21 families.^85^ It has been shown that a founder effect contributes to the recurrence of this variant.^85^ A modifier of *CLDN14*‐associated HL has been proposed but remains uncharacterized. The discovery of CLDN14's role in regulating intercellular permeability in the inner ear highlights the importance of genetic testing for *CLDN14* in individuals with HL in the moderate range.

### 5.4 GJB2

Gap junctions play a crucial role in maintaining cochlear potassium homeostasis, which is essential for hearing. *GJB2* encodes connexin 26 and is one of the most common causes of HL worldwide.^86^ Connexin 26 oligomerizes to form hexameric hemichannels called ‘connexons’ in the plasma membrane. The connexons of neighbouring cells come together to form gap junctions, which facilitate intercellular communication.^87^ Gap junctions can be homomeric or heteromeric, depending on whether they are made up of one or many different connexin proteins. This affects their ability to selectively permeate certain molecules and ions.^88^ Gap junctions allow ions, nutrients and signalling molecules with molecular weights up to 1200 Da to pass between cells.^89^ This intercellular communication is critical for the normal growth, function, and repair of the sensory epithelia of the inner ear. These processes that are disrupted through genetic mutation result in eventual cell death and HL. In the Pakistani population, the prevalence of *GJB2*‐associated HL ranges from 6.1% to 53%^90^ and it is the most common cause of NSHL in South Asia.^66^ Patients with biallelic pathogenic variants typically have congenital‐onset HL, ranging from mild to profound severity, depending on the variant, and it can be progressive in roughly half of patients.^86^ Many pathogenic variants have been identified in *GJB2*, with the most common ones in the Pakistani population being c.231G>A, p.Trp77Ter, c.71G>A, p.Trp24Ter and c.35delG, p.Gly12ValfsTer2, (NM_004004.5).^1^, ^7^, ^68^, ^91^ Among these variants, individuals homozygous for the c.35delG variant exhibit extreme phenotypic heterogeneity worldwide, ranging from mild HL to profound deafness. A founder effect has been attributed to its high prevalence. The *GJB2* c.35delG variant causes a premature stop codon in exon 2 out of 2 total exons, leading to a loss of protein function.^92^ In Pakistan, except for one variant associated with moderate–to‐severe HL, all others are associated with severe to profound deafness.^66^ To date, 29 pathogenic variants of *GJB2* have been identified in the Pakistani population.^66^

### 5.5 HGF

*HGF* (DFNB39) encodes hepatocyte growth factor (HGF), which plays a crucial role in various biological processes, including cell growth, survival, differentiation and branching morphogenesis, with implications for neuronal survival and differentiation.^93^ *HGF* dosage in the inner ear must be precisely calibrated for normal hearing. Transgenic mouse models of *Hgf* overexpression and deficiency present deafness resulting from the failure of neural crest cell migration to the intermediate cell layer of the stria vascularis, which causes thinning, reducing the endocochlear potential and hair cell loss.^33^, ^93^ While *HGF*‐associated HL is relatively prevalent in the Pakistani population, explaining 6–8% of severe to profound pre‐lingual HL, it is rarely implicated in the diagnosis of individuals with HL in other populations.^66^ In the Pakistani population, three *HGF* variants have been identified, including a synonymous variant that affects splicing and two deletions in a highly conserved region of intron 4, which is part of the 3′ untranslated region of a short *HGF* isoform.^75^ The most common variant in the Pakistani population is c.482+1986_1988delTGA (NM_000601.6) in intron 4.^33^ Mice with the 10 bp deletion corresponding to c.482+1991_2000delGATGATGAAA in human intron 4 developed defects in the stria vascularis due to failure of neural crest cell migration during development, resulting in a significantly reduced endocochlear potential.^93^ *HGF* activates the MET receptor‐mediated signalling pathway and mediates diverse downstream pathways, including those involved in the epithelial‐mesenchymal transition and the development of neural crest‐derived lineages. Overall, deleterious variants in *HGF* can result in impaired cell migration and the development of neural crest‐derived structures in the inner ear, leading to a reduced endocochlear potential and HL.

### 5.6 MARVELD2

*MARVELD2* (DFNB49) encodes a member of the marvel protein family called tricellulin that is concentrated at the tight junctions, forming part of the continuous intercellular barrier between epithelial cells. Tight junctions are multi‐protein complexes preventing leakage of solutes and water, acting as a seal between epithelial cells.^94^ Tricellular tight junctions are present in epithelial cells between supporting and hair cells, cochlear supporting cells and marginal cells of the stria vascularis.^27^ Although *MARVELD2* is ubiquitously expressed in epithelial junctions, only the inner ear appears to be affected by the genetic disruption, suggesting that genetic compensation may be present in other organ systems that are absent in the inner ear.^27^ Biallelic variants in the Occludin‐ELL domain of *MARVELD2* have been shown to cause HL in multiple Pakistani families.^95^ These variants explain between 1.5% and 2.4% of all HL in Pakistan to a moderate to profound degree. The most recurrent variant of *MARVELD2* is c.1331+2T>C (NM_001038603.3), and at least seven additional rare disease‐associated variants have also been reported in the Pakistani population.^1^ Tricellulin is essential for maintaining the integrity and stability of epithelial cells and their junctions.^27^ The occluding‐ELL domain of MARVELD2 may be necessary for the maintenance of tricellular junctions and the proper functioning of the inner ear.^96^ Deleterious variants in tricellulin lead to tight junction disorganization, resulting in severe to profound sensorineural HL.^27^

### 5.7 MYO7A

*MYO7A* (DFNB2) encodes an unconventional myosin that plays a crucial role in maintaining the mechanical stability of the hair bundle. The hair bundle is the sensory structure located on the surface of hair cells in the inner ear, acting as a mechanotransducer that transforms sound waves or orientation information into electrical signals, which the brain interprets (Houdusse & Titus, 2021).^97^ This is achieved through the transportation of extracellular stereocilia links along actin filaments^98^ at the upper tip‐link density and ankle link region of the stereocilia by *MYO7A*, which is essential for the cohesion of the hair bundle.^99^ MYO7A co‐localizes with several other proteins at the upper tip‐link density and ankle link region of the stereocilia, including CDH23, USH1C, USH1G, ADGRV1 and USH2A, which are integral for the proper functioning of the hair bundle (Houdusse & Titus, 2021).^97^ Moreover, independent of molecular trafficking, MYO7A exerts force at the upper tip‐link density region and tensions the MET complex, further highlighting its important role in hearing.^100^ In addition to its role in the inner ear, *MYO7A* is also expressed in retinal pigment epithelial cells where it is required for functional RPE65, a key protein in the retinoid cycle.^101^ *MYO7A* is also associated with Usher syndrome type I, an autosomal recessive condition characterized by deafness, vestibular impairment and retinitis pigmentosa. Biallelic variants in *MYO7A* account for 29% to 50% of all USH1 cases globally, making it the most common cause of this condition.^102^ Moreover, at least 11% of moderate to severe pre‐lingual SNHL in the Pakistani population is due to *MYO7A* disruption^42^ with at least 59 variants reported.^66^ Among these variants, c.397dupC, p.His133ProfsTer7 and c.470G>A, p.Ser157Asn (NM_000260.3) are the most prevalent in the Pakistani population.^68^ These variants exhibit founder effects that are not seen in other populations.^68^

### 5.8 MYO15A

Myosin XVa, a product of *MYO15A*, belongs to the unconventional myosin superfamily and is essential for the elongation of stereocilia in cochlear sensory and vestibular hair cells. The growth of the hair bundle is facilitated by the transportation of whirlin and Eps8 to the tip of stereocilia to form the stereocilia tip complex by Myosin XVa, which can help in the conversion of microvilli into fully mature stereocilia.^103^ MYO15A is involved in the regulation of actin and the transportation of elongation complexes at the distal stereocilia tip as well as other cargoes for actin.^99^ Notably, the motor and tail domains of myosin XVa have been identified as crucial for normal auditory structure and function.^104^ In Pakistani families, at least 49 *MYO15A* variants have been linked to 5–13% of progressive severe to profound bilateral SNHL.^66^ The majority of the variants in this gene have been found in affected individuals from one to three Pakistani families, except for two variants, c.6589C>T, p.Gln2197Ter and c.8158G>C, p.Asp2720His (NM_016239.3) found in deaf members of four families each.^59^ *MYO15A* variants significantly impact the motor domain, leading to dysfunction causing shorter stereocilia with an ectopic staircase structure, a condition associated with severe deafness.^105^

### 5.9 MSRB3

*MSRB3* (DFNB74) encodes methionine sulfoxide reductase B3 (MSRB3) that plays a critical role in repairing oxidatively damaged proteins by catalysing the stereo‐specific reduction of methionine‐R‐sulfoxides to methionine.^37^ *Msrb3* localizes to the base of the stereocilia on the apical surface of hair cells.^106^ Studies on the *Msrb3* ^*−/−*^ mouse model have shown that MSRB3 is essential for the maturation and/or maintenance of stereociliary bundles since these mice exhibit progressive degeneration of the stereociliary bundles and apoptosis of hair cells.^106^ Moreover, genetic disruption of *Msrb3* is more likely to be due to degeneration rather than abnormal development, since the hair cells of KO mice develop normally and have functional mechanotransduction channels until at least P3.^106^ The c.265T>G, p.Cys89Gly and c.55C>T, p.Arg19Ter (NM_001031679.2) *MSRB3* variants are associated with deafness in six and two unrelated DFNB74 Pakistani families, respectively.^37^ A homozygous missense variant c.20T>G, p.Leu7Arg was identified in 2014 in one Pakistani family, and a homozygous splice variant, c.412‐1G>A was described in another Pakistani family in 2019.^66^ These variants are associated with severe to profound HL. Among the four variants reported in individuals with genetic ancestry from Pakistan so far, p.Cys89Gly is the most recurrent variant. This *MSRB3* cysteine residue, conserved in orthologs from yeast to humans, is involved in structural zinc binding. In vitro, this non‐synonymous substitution (p.Cys89Gly) reduced zinc binding and MSRB3 enzymatic activity.^37^

### 5.10 OTOF

*OTOF* (DFNB9) encodes otoferlin, a protein that is essential for normal hearing. Otoferlin plays a crucial role in various functions of synaptic signalling, including sensing of pre‐synaptic Ca^+2^ for exocytosis following IHC depolarization, priming and replenishment of the synaptic vesicles to maintain fast neurotransmitter release and coupling of exocytosis‐endocytosis.^107^, ^108^ Most individuals exhibit profound pre‐lingual deafness due to biallelic pathogenic or likely pathogenic *OTOF* variants. The affected individuals show isolated failure in synaptic transmission, and their otoacoustic emissions are usually initially unaffected, with healthy outer hair cells, particularly in younger individuals. The HL resulting from *OTOF*‐associated deficits is commonly known as auditory synaptopathy.^109^, ^110^ *OTOF* variants account for 3.1%–4% of pre‐lingual moderate or profound HL in Pakistan. The first variant described, a nonsense variant c.4491T>A, p.Tyr1497Ter (NM_001287489.2) in *OTOF*, was identified in four independent Lebanese families by a candidate gene approach.^111^ Since then, at least 23 *OTOF* variants associated with moderate–to‐severe or profound HL have been identified in hearing‐impaired individuals from Pakistan.^32^, ^84^ Among these, one of the most common is the c.2122C>T, p.Arg708Ter^66^ variant.^112^ This variant has been detected in both homozygous and compound heterozygous states in individuals with prelingual NSHL or auditory neuropathy in a large population cohort.^32^

### 5.11 SLC26A4

*SLC26A4*, also known as pendrin, is a protein that belongs to the solute carrier family 26 and functions as an anion exchanger, transporting negatively charged ions such as chloride, iodide and bicarbonate across cell membranes. This protein is crucial for the development of the cochlea and vestibular duct's bony snail shape structure.^113^ Variants in *SLC26A4* (DFNB4) have been linked to both ARNSHL and Pendred syndrome.^114^ While individuals with isolated HL may experience bilateral, profound SNHL, those with Pendred syndrome may have concomitant HL, enlarged vestibular aqueduct and abnormal iodine organification.^115^ Approximately 12.4% of HL cases in the Pakistani community can be attributed to *SLC26A4*.^66^ While the majority of the identified variants are described as non‐syndromic, especially in young individuals, the presence of Pendred syndrome may be frequently overlooked. *SLC26A4* has several recurring variations. In the majority of affected individuals, HL is associated with three variants: c.269C>T, p.(Ser90Leu), c.716 T > A, p.(Val239Asp), and c.1337A > G, p.(Gln446Arg) (NM_000441.1). A founder effect has been shown for their recurrence.^84^, ^116^

### 5.12 TMC1

Transmembrane channel‐like protein isoform‐1 (*TMC1*) plays a crucial role in the auditory system as it forms an ion‐conducting pore of the MET channel in auditory hair cells.^117^ Over the past 5 years, *TMC1* has emerged as a leading contender for the MET channel in auditory hair cells of the inner ear. Hair cells convert acoustic and vestibular stimuli into electrical responses through the activation of MET.^118^ *TMC1* is thought to have a six‐transmembrane domain structure similar to several other ion‐channel subunits and is transported to the tips of the stereocilia in the sensory hair bundle where the MET channel is located.^119^ *TMC1* variants associated with human deafness result in loss of typical MET currents and hair cell senescence, causing cell death. Valuable insights into the pathogenesis of *TMC1*‐associated deafness have been gained from studies of mutant mice.^120^ Variants in *TMC1* cause both dominant (DFNA36) and recessive (DFNB7/11) forms of NSHL. Both progressive postlingual and pre‐lingual profound HL have been associated with *TMC1* variants. In Pakistan, at least 32 different *TMC1* variants account for 6.4% of recessively inherited HL cases.^66^ The most frequently occurring *TMC1* variant in the Pakistani population is c.100C>T, p.Arg34Ter (NM_138691.2), which has been reported in two studies.^84^, ^121^ This common pathogenic nonsense variant is a likely founder mutation in North African and Middle Eastern populations.^7^, ^17^

### 5.13 TMPRSS3

Genetic mutation of type II transmembrane serine protease (*TMPRSS3*) causes variable HL. *TMPRSS3* has four functional domains that include an N‐terminal transmembrane domain, a low‐density lipoprotein receptor A domain, a scavenger receptor cysteine‐rich domain and a C‐terminal serine protease domain.^122^ *TMPRSS3* is expressed in various components of the developing inner ear, such as the stria vascularis, spiral ganglion neurons,^123^ IHCs and cochlear aqueduct of the foetal cochlea and is critical for their normal development and maintenance^124^ Additionally, cytoplasmic domains of *TMPRSS3* raise the possibility that they may participate in intracellular signal transduction.^122^ Biallelic variants in *TMPRSS3* are known for causing different types of HL with variable onset. DFNB10 is characterized by congenital or childhood‐onset bilateral profound HL,^125^ whereas DFNB8 is associated with a milder postlingual progressive HL,^126^ both of which are caused by variants in *TMPRSS3* (DFNB8/10). In Pakistan, *TMPRSS3* variants are associated with stable, moderate–to‐severe or profound HL and contribute up to 4% of the prevalence of HL.^66^ Other frequently occurring variants include c.323‐6G>A (splicing) and c.1219T>C, p.Cys407Arg.^84^ The recurring homozygous c.1219T>C, p.Cys407Arg (NM_024022.2) variant has been identified in 20 Pakistani families.^127^ Functional studies show that the variant protein has defective protease activity compared to the wild‐type (Lee et al., 2003) as well as a failure to undergo proteolytic cleavage and activate the epithelial sodium channel (ENaC) in vitro.^124^, ^128^ In silico analysis also supports that this missense variant has a negative effect on protein structure/function. There are 14 other rare variants in this gene that contribute to NSHL in the Pakistani population.

## 6 CONCLUSION

This review explores the clinical and genetic complexities of HL, defining and describing key characteristics, terminology and genetics. After 25 years of engaging the Pakistani population, researchers have uncovered variants in 51 genes and made significant progress in understanding the most frequently implicated ARNSHL genes. Of these, 39 genes causally related to HL were discovered through the use of gene mapping methodologies and sequencing strategies in consanguineous Pakistani families. This aggregated knowledge has established 13 being the most commonly involved in the molecular diagnosis of Pakistani patients (*CDH23*, *CIB2*, *CLDN14*, *GJB2*, *HGF*, *MARVELD2*, *MYO7A*, *MYO15A*, *MSRB3*, *OTOF*, *SLC26A4*, *TMC1* and *TMPRSS3*). We detailed their purpose and highlighted important variants from the Pakistani community's standpoint. For a detailed molecular understanding of these ARNSHL genes, we also categorized enriched GO terms and shared pathways using Metascape.

Engaging the Pakistani community has been fundamental in advancing gene discovery. The high prevalence of consanguinity and congenital HL, a predominantly recessive trait, has provided fundamental insights into the genes and variants underlying HL over several decades. This has amplified the global knowledge base, providing valuable information for the selection of therapeutic targets and improving genetic diagnoses. However, HL continues to impose a significant burden on affected individuals, necessitating the discovery of new strategies for more precise diagnosis, alleviation, and treatment. Much work remains to achieve a comprehensive understanding of all genes and variants causing HL.

## AUTHOR CONTRIBUTIONS

**Madiha Shadab:** Conceptualization (equal); data curation (equal); investigation (equal); methodology (equal); project administration (equal); resources (equal); validation (equal); visualization (equal); writing – original draft (equal); writing – review and editing (equal). **Ansar Ahmed Abbasi:** Methodology (equal); project administration (equal); supervision (equal); writing – review and editing (equal). **Ahsan Ejaz:** Data curation (equal); resources (equal). **Afif Ben‐Mahmoud:** Methodology (equal); writing – review and editing (equal). **Vijay Gupta:** Formal analysis (equal); writing – review and editing (equal). **Hyung‐Goo Kim:** Funding acquisition (equal); methodology (equal); supervision (equal); visualization (equal); writing – review and editing (equal). **Barbara Vona:** Conceptualization (equal); funding acquisition (equal); investigation (equal); methodology (equal); project administration (equal); validation (equal); writing – original draft (equal); writing – review and editing (equal).

## FUNDING INFORMATION

Barbara Vona is funded by the German Research Foundation DFG VO 2138/7‐1 grant 469177153. Hyung‐Goo Kim is funded by IGP5 funding from the Qatar Biomedical Research Institute at Hamad Bin Khalifa University.

## CONFLICT OF INTEREST STATEMENT

The authors confirm that there are no conflicts of interest.

## Supporting information


Table S1.

## Data Availability

Data sharing not applicable to this article as no datasets were generated or analysed during the current study.
